# Combination therapy of human umbilical cord mesenchymal stem cells and FTY720 attenuates acute lung injury induced by lipopolysaccharide in a murine model

**DOI:** 10.18632/oncotarget.20491

**Published:** 2017-08-24

**Authors:** Zili Zhang, Wenfei Li, Zhizhi Heng, Jing Zheng, Puyuan Li, Xin Yuan, Wenkai Niu, Changqing Bai, Huiying Liu

**Affiliations:** ^1^ Department of Respiratory and Critical Care Diseases, 307th Hospital of PLA, Beijing 100071, China; ^2^ Anhui Medical University, Hefei 230032, China; ^3^ School of Aerospace Medicine, Fourth Military Medical University, Xi’an 710032, China; ^4^ Beijing Geriatric Hospital, Beijing 100095, China

**Keywords:** acute lung injury, lipopolysaccharide, hUC-MSCs, FTY720, Sphingosine-1-phosphate

## Abstract

ALI/ARDS remain the main reason of morbidity and mortality in the critically ill. Studies have indicated that human umbilical cord mesenchymal stem cells (hUC-MSCs) can be useful in the treatment of ALI/ARDS. Sphingosine-1-phosphate (S1P) and its analog FTY720 significantly reduce lipopolysaccharide (LPS)-induced lung edema and inflammatory lung injury. This study aimed to assess the therapeutic effects of hUC-MSCs combined with FTY720 in an LPS-induced murine model of ALI. Eight-week-old female C57BL/6 mice were divided into a normal control group, an LPS group, an hUC-MSC group, an FTY720 group, and an hUC-MSCs+FTY720 group randomly. At 24 hours post injury, mice were administrated hUC-MSCs via the tail vein and/or intraperitoneally injected with FTY720. We assessed histopathology and histologic scores, lung wet/dry weight ratio, micro-CT scans, and total protein in the bronchoalveolar lavage fluid (BALF), as well as cytokines in the BALF at 48 h post injury. All treatment groups showed higher survival rates and attenuated lung injuries. The hUC-MSCs+FTY720 group yielded better results than hUC-MSCs or FTY720 alone. While the underlying mechanism requires further study, we anticipate that combination therapy of hUC-MSCs and FTY720 could be an effective strategy for ALI.

## INTRODUCTION

Acute lung injury (ALI) and acute respiratory distress syndrome (ARDS) both can result in acute respiratory failure, and are remain the main reasons of death of critically ill patients [1]. These syndromes are induced by various clinical diseases and are characterized by serious intractable hypoxemia and increased inspired oxygen fraction [2]. Most treatments to improve hypoxemia in ARDS aim to improve aeration and ventilation [3]. Unfortunately, because of the short of effective therapy, the mortality is extremely high [4]. Lipopolysaccharide (LPS) is widely used for the establishment of ALI models. Recently, transplanting human mesenchymal stem cells or endothelial progenitor cells, was pointed out to alleviate ALI and decrease mortality induced by LPS [5–7]. In several researches [8, 9], MSCs are able to improve pathological damage, reduce inflammation, and even reduce mortality in a mouse model of ALI/ARDS. These researches manifest that hUC - MSCs could be a new method of treatment for ALI/ARDS [7].

Sphingosine-1-phosphate (S1P) is a kind of natural bioactive sphingolipid. It acts extracellularly via the G protein-coupled S1P_1–5_ as well as intracellularly on different aims, and mainly exists in tissues and plasma [10]. Some researches have indicated that S1P is a potent angiogenic factor. In preclinical animal models of ALI it can enhance lung endothelial cell integrity and is an inhibitor of vascular permeability and alveolar flooding [11–13]. In addition to S1P, S1P analogs, such as FTY720, provide a potential treatment in lung injury of murine models [14]. However, FTY720 decreased pulmonary function and increased the rate of dyspnea in a recent multiple clinical trial, not vice versa [15]. Thus, new mechanism findings are needed to lead to innovation strategies and treatments of lung injury [14].

In our previous study [16], we found that TNF-α expression was down-regulated more significantly if hUC-MSC was used in combination with S1P in a human pulmonary artery endothelial cells(HPAEC) model of acute injury. The combined effect mainly worked on S1PR2, S1PR3 and SphK2. When hUC-MSCs were combined with S1P, it also showed that the selectivity of S1P receptors was increased and the homeostatic control of S1P concentration was improved by regulating expressions of S1P metabolic enzymes [16].

ALI is an intricate pathophysiological process, and therapy targeted singly may not be the most effective. Therefore, we hypothesized that combination of transplantation of hUC-MSCs with FTY720 could reduce LPS-induced ALI in mice. In this study, we examined survival, histology, and pulmonary inflammation in ALI mice treated with hUC-MSCs, FTY720, or the combination of both.

## RESULTS

### Combinatorial treatment superiorly attenuates LPS-induced lung injury

**Figure 1:**
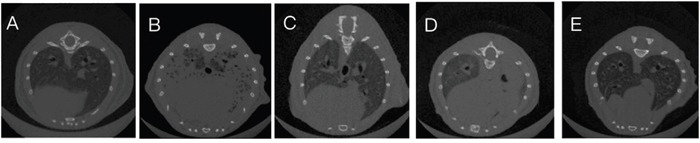
CT scans of lung tissues at 48 h after LPS challenge **(A)** Control, **(B)** LPS+PBS, **(C)** LPS+hUC-MSCs, **(D)** LPS+FTY720, **(E)** LPS+ hUC-MSCs+ FTY720.

CT scans of the mice lungs of all treatment groups at 48 h after LPS administration were generated. The results showed that compared with the LPS+PBS group, mice in LPS+hUC-MSCs, LPS+FTY720, and LPS+hUC-MSCs+FTY720 groups showed clearly attenuated inflammation in the lungs after LPS challenge (Figure 1).

**Figure 2:**
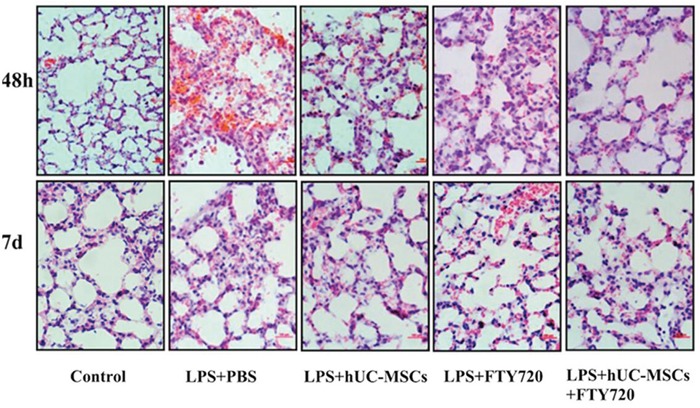
Histopathological images of lung tissue sections at 48 h and 7 days after LPS challenge (magnification, 200×)

**Figure 3:**
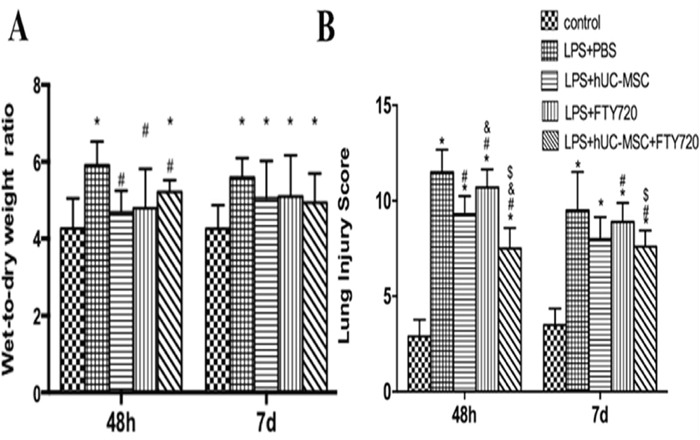
Quantification of lung edema and injury in LPS-challenged mice **(A)** Lung edema was determined as the lung wet/dry weight ratio. Results are shown for samples taken at 48 h and 7 days after LPS challenge. **(B)** Lung injury scores show an obvious decrease in the severity of lung injury in the LPS+hUC-MSCs and LPS+FTY720 groups, and especially, in the LPS+hUC-MSCs+FTY720 group. Data are represented by the mean ± SD, n = 10 at each time point for each group. *P < 0.05 vs. control; ^#^*P* < 0.05 vs. LPS+PBS; ^&^*P* < 0.05 vs. LPS+ hUC-MSCs; ^$^*P* < 0.05 vs. LPS+FTY720.

Left lung tissue sections were evaluated for edema, alveolar and interstitial inflammation, hemorrhage, and hyaline membrane formation. The sum of these scores was as a result of total lung injury score [18]. We selected each slide randomly and chosed high-power fields (200×) to be analyzed, and the two investigators were blinded to the treatment groups (Figure 2). At 48 h, the lung tissues of mice were found with inflammatory cell infiltration, hemorrhage, alveolar exudates, and edema. Also the injury scores were increased (Figure 3B). However, these pathological changes and injury scores were diminished in the LPS+hUC-MSCs, LPS+FTY720, and LPS+hUC-MSCs+FTY720 groups as compared to the LPS+PBS group at this time point (*P* < 0.05). The effect was more significant in the LPS+hUC-MSCs+FTY720 group than in the other groups (*P* < 0.05). At day 7, the histopathologic characteristics and injury scores were recovered to nearly normal in the control and treatment groups.

The wet/dry lung-weight ratio was determined to assess edema (Figure 3A). Lung edema was significantly alleviated at 48 h in the LPS+hUC-MSCs, LPS+FTY720, and LPS+hUC-MSCs+FTY720 groups as compared to the LPS+PBS group (*P* < 0.05), while there was no difference at 7 days.

### hUC-MSCs, FTY720, and combinatorial treatment groups had higher survival rate

**Figure 4:**
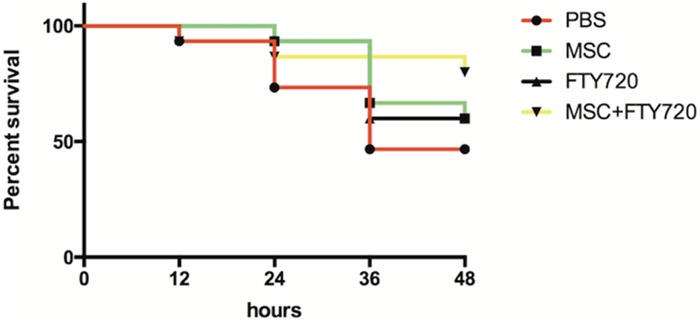
Mouse survival rates (n = 15 for each group) at 48 h after LPS challenge using Kaplan–Meier analysis followed by a log-rank test The survival of mice in the LPS+hUC-MSCs+FTY720 group was higher than that in other experimental groups, but there were not statistically significant in the differences (*P* = 0.07, LPS+hUC-MSCs+FTY720 vs. LPS+PBS; *P* = 0.29 LPS+hUC-MSCs+FTY720 vs. LPS+hUC-MSCs; *P* = 0.31 LPS+ hUC-MSCs+FTY720 vs. LPS+FTY720).

Four groups of mice (n = 15 per group) were used for survival study. After the treatments, the mice recovered soon, and mortality was observed and recorded to 48 h. hUC-MSCs, FTY720, and combinatorial treatment groups had higher survival rate compared with PBS group at 48 h. The survival rate of combination therapy group of FTY720 and hUC-MSCs was higher than other groups at 48 h, although all the differences were not statistically significant (Figure 4).

### Combinatorial treatment superiorly attenuates vascular permeability and lung tissue inflammation

**Figure 5:**
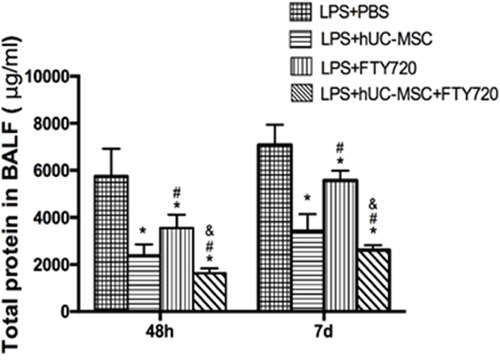
Total protein concentration in the BALF at 48h and 7 days after LPS administration Data are represented by the mean ± SD (n = 10 at each time point for each group). *P < 0.01 vs. LPS+PBS; ^#^*P* < 0.01 vs. LPS+hUC-MSCs; ^&^*P* < 0.01 vs. LPS+FTY720.

**Figure 6:**
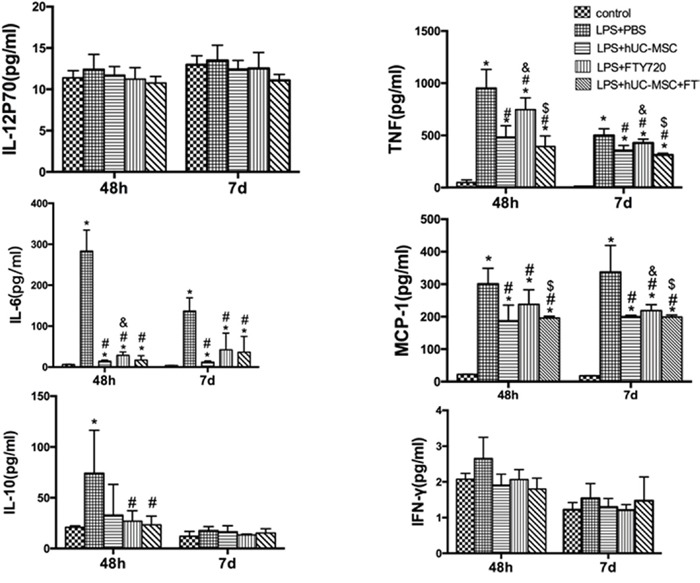
Inflammatory cytokine levels in the BALF at 48 h and 7 days after LPS administration BALF was obtained for measuring these indicators using mouse inflammation kits. Data are represented by the mean ± SD (n = 6 at each time point for each group). *P < 0.01 vs. control; ^#^*P* < 0.05 vs. LPS+PBS; ^&^*P* < 0.05 vs. LPS+hUC-MSCs; ^$^*P* < 0.05 vs. LPS+FTY720.

Compared to the LPS+PBS group, total protein in BALF was significantly reduced in the LPS+hUC-MSCs, LPS+FTY720, and LPS+hUC-MSCs+FTY720 groups (*P* < 0.01) at 48 h and 7 days (Figure 5). The levels of inflammation marker MCP-1, IL-10, IL-12p70, TNF, IL-6, and IFN-γ were measured in the BALF of mice at 48 h and 7 days after treatment for each group(Figure 6). TNF, IL-6, and MCP-1 were obviously lower in the three treatment groups than in the LPS+PBS group (*P* < 0.01). The decreases in TNF and MCP-1 were greater in the LPS+hUC-MSCs+FTY720 than in the two other groups (*P* < 0.01). However, no differences were founded in IL-10, IL-12p70 and IFN-γ between the three treatments.

## DISCUSSION

Previous studies have shown that hUC-MSCs home to injured lungs and differentiate into pulmonary epithelial cells and thus, can be regardred as a potential treatment for ALI [17]. Up to now, the mechanisms responsible for the treatment of MSCs in ALI are not entirely clear. Their capacity for pluripotent and secretion of multiple paracrine factors may be involved in these effects. Our previous study found that hUC-MSCs could impact on multiple S1P related genes simultaneously as a potential therapy. The expression regulation result of related genes was not just the superposition of each other, but more obvious outcome, while hUC-MSCs were combined with S1P [16]. To further confirm the therapeutic effect of hUC-MSCs combined with SIP in the therapy of ALI, we used S1P analog FTY720 combined with hUC-MSCs to evaluate the therapeutic efficacy in LPS induced injury mice.

Our study showed that combination therapy of hUC-MSCs with FTY720 significantly attenuates ALI after LPS challenge as compared to each treatment alone. This suggests that FTY720 might help to improve the therapeutic effect of hUC-MSCs in ALI.

The primary target of this work was to investigate the therapeutic effects of combination therapy of hUC-MSCs and FTY720 in ALI induced by LPS in mice. CT scans, lung histology, and injury scores suggested that the effects of the combination therapy were greater than those of hUC-MSCs or FTY720 alone. The major finding of this study was that combination therapy significantly improved the extent of lung injury, which was assessed quantitatively by the use of lung injury scores as described previously [18]. Increased capillary permeability in lung is considered to be a critical factor that protein-rich edema fluid infiltrate into the alveolar space [19]. Many researches have showed that MSCs and FTY720 treatment significantly reduce the lung wet/dry weight ratio and the protein in BALF [8, 20]. Consistently, our study indicated that lung edema and total protein in the BALF were obviously lowered after treatment for each group, with the combination therapy showing superior effect. ALI is an uncontrollable lung inflammation caused by a great quantity of inflammatory cells and cytokines [9]. The pro-inflammatory mediators TNF, MCP-1, and IL-6 were increased, which is evidence of acute systemic inflammatory response caused by LPS. Earlier reports suggested that LPS challenge increases IL-6 secretion and pulmonary leakage [21]. The decrease in anti-inflammatory could be one of the key mechanisms of the treatment.

Some studies reaffirmed the conclusion that an antagonistic relationship existed between S1PR1 and S1PR2 in the vascular endothelium during tissue injury and disease [22]. In an acute injury model of HPAEC, it found that the expressions of S1PR1-3 all increased. However, the expression of S1PR1 was not obviously changed while the expressions of S1PR2 and S1PR3 decreased significantly when hUC-MSCs were given [16]. We speculate that if hUC-MSCs could up-regulate the S1P receptors selectivity *in vivo*, so as to enhance the endothelial barrier function.

Clinical studies have indicated that no single target or drug can reverse the serious pathological damage caused by ARDS/ALI and achieve the expected treatment outcome rapidly. Combination therapy of FTY720 with hUC-MSCs may be a new approach to treatment for reducing the high mortality and morbidity caused by ALI in humans.

## MATERIALS AND METHODS

### Animals

8 to 12 weeks female C57BL/6 mice were purchased from Beijing HFK Bioscience Co. (Beijing, China). They were keeped in individual cages with free access to water and food (Laboratory Animal Center of the Academy of Military Medical Sciences, Beijing, China). All animal experiments conformed to NIH guidelines (Guide for the care and use of laboratory animals) (NIH Publication No. 85-23, revised 2011). We divided mice into 5 treatment groups randomly: a normal control (control) group, a PBS control (LPS+PBS) group, an hUC-MSC treatment (LPS+hUC-MSCs) group, an FTY720 treatment (LPS+FTY720) group, and an hUC-MSC plus FTY720 treatment (LPS+hUC-MSCs+FTY720) group. The mice were all euthanized finally.

### Establishment of the ALI mouse model

To induce ALI, we anesthetized mice with pentobarbital sodium and then intratracheally instilled with *Escherichia coli* O55:B5 LPS (10 mg/kg body weight, 50 μl; Sigma, St. Louis, MO). Mice were then recovered again as before, and survival was noted. The mice were sacrificed for assessment after 48 hours or 7 days and samples were collected according to the different experiments.

### hUC-MSCs and FTY720 treatments

Mice were given an injection of PBS or hUC-MSCs (2 × 10^5^ cells) in a 200-μl volume via the tail vein at 24 h after the LPS challenge. hUC-MSCs were obtained from the 307-IVY Translation Medicine Center (Laboratory of Oncology, Affiliated Hospital of Academy of Military Medical Sciences, Beijing, China). A single intraperitoneal injection of FTY720 reduces murine lung injury obviously and low concentrations of FTY720 (0.1 mg/kg) significantly reduce lung permeability [11, 23]; therefore, mice in the LPS+FTY720 and LPS+hUC-MSCs+FTY720 groups were given an intraperitoneal injection of FTY720 (0.1 mg/kg, 200 μl; Sigma, St. Louis, MO) at 24h after LPS administration.

### Sample acquisition

Lungs were harvested at 48 h and 7 days after LPS administration. Left lung lobes were fixed in 10% formalin, paraffin-embedded, and sectioned at 5-μm thickness. BAL fluid (BALF) was collected at 48 h by flushing 1 ml of ice-cold PBS back and forth three times through a tracheal cannula with a 20-gauge catheter to assess concentrations of protein and inflammatory factors as indexes of lung permeability (injury). BALF was centrifuged at 1,500 ×*g* for 10 min at 4°C and stored at –80°C until analysis.

### Histological analysis

Lung tissue sections were stained with hematoxylin and eosin and mice were examined by micro-CT (PerkinElmer-Caliper LS, Co., Boston, MA) for lung morphology. A scoring system to grade the degree of lung injury was used, based on the following histologic features: edema, hyperemia and congestion, neutrophil margination, and tissue infiltration. Edema, alveolar and interstitial inflammation, hemorrhage, and hyaline membrane formation were each scored using a 0 to 4 point scale (no injury, 0; injury in 25% of the field, 1; injury in 50%, 2; injury in 75%, 3; and injury throughout the field, 4). The sum of these scores was as a result of total lung injury score [18].

### Lung wet/dry weight ratio measurement

After the mice were sacrificed, right lungs were excised, blotted dry, and weighed. Lung dry weights were recorded after the samples were dried in an oven at 80°C for 12 h. The lung wet/dry weight ratio was used to assess the degree of pulmonary edema.

### Total protein and inflammatory factors in the BAL

Total protein concentration in the BALF was examined by a standard BCA kit (BD Biosciences, San Jose, CA). Inflammatory markers IL-12p70, IL-10, IL-6, TNF, MCP-1, and IFN-γ in the BALF were examined by BD Cytometric Bead Array (CBA) mouse inflammation kit (BD Biosciences) per the manufacturer's instructions.

### Statistical analysis

The data are expressed as the mean ± SD. Kaplan–Meier analysis followed by a log-rank test was used to analyse survival rates. Group means were compared using a *t*-test. All data were analyzed using SPSS version 17.0 and GraphPad Prism 5. A *P*-value < 0.05 was considered statistically significant.

## Abbreviations

ALI: acute lung injury
ARDS: acute respiratory distress syndrome
hUC-MSCs: human umbilical cord mesenchymal stem cells
S1P: Sphingosine-1-phosphate
S1PR: Sphingosine-1-phosphate receptor
LPS: lipopolysaccharide
BALF: bronchoalveolar lavage fluid
IL: interleukin
TNF-α: tumor necrosis factor-α
CT: computed tomography
PBS: phosphate buffer saline
MCP-1: monocyte chemotactic protein-1
IFN: interferon
CBA: cytometric bead array
HPAEC: human pulmonary artery endothelial cells
